# Clinical features, microbiology, and management of pediatric brainstem abscess

**DOI:** 10.1007/s00381-020-04835-9

**Published:** 2020-07-30

**Authors:** Łukasz Antkowiak, Monika Putz, Marek Mandera

**Affiliations:** Department of Pediatric Neurosurgery, Medical University of Silesia, Ul. Medyków 16, 40-752 Katowice, Poland

**Keywords:** Brainstem abscess, *Streptococcus intermedius*, Management, Diagnosis

## Abstract

**Purpose:**

Brainstem abscess is a rare condition accounting for merely 1% of brain abscesses incidence in the pediatric population. This study aimed to present a single patient with a pontine abscess and review the literature to highlight clinical features, diagnosis, and management of brainstem abscess.

**Methods:**

The PubMed database was screened for English-language articles concerning pediatric brainstem abscess. We, therefore, identified 22 publications, which concisely depict 23 cases. Our study reports on the 24th pediatric patient diagnosed with that entity. All included reports were analyzed in terms of clinical presentation, diagnosis, management, and outcomes of described patients.

**Results:**

There was slight women predominance (15:9), with a mean age of occurrence 6.4 years, ranging from 7 months to 16 years. Pons was the most common location of brainstem abscess, occurring in 75% of patients. Clinically, they mostly presented with cranial nerves palsy (79.2%), hemiparesis (66.7%), and pyramidal signs (45.8%). The classic triad of symptoms, including fever, headache, and the focal neurologic deficit was present in 20.8% of patients. Positive pus cultures were obtained in 61.1%. Streptococci and Staphylococci were the most frequently identified pus microorganisms. Outcomes were satisfactory, with a 79.2% rate of general improvement.

**Conclusions:**

Neurosurgical aspiration is a safe and beneficial therapeutic method. It should always be considered and should promptly be performed when the conservative treatment is not successful and clinical deterioration occurs. Prognosis in pediatric brainstem abscess is generally favorable. Most patients recover with minor neurologic deficits or improve completely.

## Introduction

Brain abscess is defined as a collection of pus in the brain parenchyma. It appears typically in a devitalized area or affected by poor microcirculation [1]. Its incidence is estimated between 0.4 and 0.9 per 100,000 people per year [2]. Nathoo et al. report that 42% of brain abscesses appear in the pediatric population [3]. Their typical location includes frontal, temporal, parietal lobes, and cerebellum. Merely 1% of all brain abscesses are found within the brainstem [4, 5]. To date, 23 childhood brainstem abscesses were described in the literature, and we report on the 24th case [5–26]. Typical triad of symptoms, including fever, headache, and the focal neurologic deficit, is being found in merely up to 28% of patients diagnosed with brain abscess [27]. Clinical presentation varies depending on abscess location in the brain. In up to 35% of patients, the primary source of infection cannot be determined; therefore, they are classified as cryptogenic [4, 28, 29]. Streptococci and Staphylococci are the most frequently isolated microorganisms, followed by anaerobes and Gram-negative enteric bacteria [30, 31]. We report on a single pediatric patient diagnosed with a pontine abscess caused by *Streptococcus intermedius* of undetermined origin. Furthermore, we reviewed English-language literature to discuss the diagnosis and management of brainstem abscess in children.

### Historical background

The first pediatric patient diagnosed with brainstem abscess was presented in 1974 by Danziger et al. [8]. To date, 23 cases of that entity were described in world literature, and we present the 24th pediatric patient. The mean age of occurrence in cerebral abscesses ranges between 4 and 7 years [28]. Considering brainstem abscesses, the mean age of occurrence during childhood was 6.4 years, ranging from 7 months to 16 years. Pontine location was the most common, found in eighteen patients (75%); ten children had a midbrain location (41.7%), whereas only four patients (16.7%) were diagnosed with an abscess in the medulla oblongata (Table 1). Abscess involving more than one part of the brainstem was seen in 7 patients (29.2%); thereof, only one patient presented with entire brainstem neuroaxis affected (midbrain, pons, and medulla).

**Table 1:** General information on pediatric patients diagnosed with brainstem abscess [5–26]

Feature	Number (%)
Demographic features	
Male	9 (37.5)
Female	15 (62.5)
Mean age	6.4 years (7 months–16 years)
Abscess location	
Midbrain	10/24 (41.7)
Pons	18/24 (75.0)
Medulla oblongata	4/24 (16.7)

In the pediatric population, factors predisposing to brain abscess development were found in 56–86% of patients. Amidst underlying infectious risk factors are sinusitis, periorbital and orbital cellulitis, otitis media, meningitis, and mastoiditis [4, 27, 32, 33]. No studies considering pediatric brainstem abscesses risk factors were conducted due to low incidence accounting for less than 1% of cerebral abscesses [5]. Nevertheless, our study shows that among infectious conditions, otogenic etiology was the most frequently appearing, followed by dental source and meningitis. Sinusitis was not identified in any patient. It can be easily explained since most of the sinusitis-related abscesses localize in the frontal lobes, which is also the most commonly affected cerebral location for abscesses [27]. Brainstem abscesses tend to spread through a hematogenous route from distant locations such as the lungs or heart, alternatively from primary otogenic infection [32]. Among underlying non-infectious primary predisposing conditions are congenital heart disease, immunodeficiency, and neurosurgical intervention [33]. Six brain abscesses (25%) were associated with underlying congenital heart disease, being also the most commonly identified etiology. The majority of abscesses located in the brainstem are defined as cryptogenic, with no infection source found on diagnostics. To date, nine out of 24 cases, accounting for 37.5%, were described as cryptogenic (Table 2). Literature estimates that incidence at about 15–35% of brain abscesses [34, 35].

**Table 2:** Clinical presentation and etiology of brainstem abscess [5–26]

Feature	Number (%)
Symptoms	
Fever	13/24 (54.2)
Headache	12/24 (50.0)
Vomiting	6/24 (25.0)
Altered level of consciousness	10/24 (41.7)
Focal neurologic deficit	21/24 (87.5)
Classic triad (fever, headache, focal neurologic deficit)	5/24 (20.8)
Neurologic deficit	
Hemiparesis	16/24 (66.7)
Cranial nerve palsy	19/24 (79.2)
Meningeal signs	0
Papilledema	2/24 (8.3)
Anisocoria	2/24 (8.3)
Seizures	1/24 (4.2)
Upward/downward gaze palsy	5/24 (20.8)
Ptosis	6/24 (25.0)
Ataxia	5/24 (20.8)
Strabismus	9/24 (37.5)
Dysphagia	4/24 (16.7)
Dysarthria	3/24 (12.5)
Aphasia	2/24 (8.3)
Pyramidal signs	11/24 (45.8)
Predisposing factors	
Congenital heart disease	6/24 (25.0)
Dental infection	2/24 (8.3)
Otitis	3/24 (12.5)
Mastoiditis	0
Sinusitis	0
Pulmonary	2/24 (8.3)
Meningitis	1/24 (4.2)
Cryptogenic	9/24 (37.5)
Melioidosis	1/24 (4.2)

### Clinical presentation

Classic brain abscess-related triad of symptoms including headache, fever, and focal neurologic deficit appears in merely 9–28% of pediatric patients [27]. According to the 24 described brainstem abscesses, that symptomatology occurred in 5 patients (20.8%). Typically, headache is the most common symptom and fever being the second accompanying brain abscess [29]. In our series, the headache was present in 12 (50%) and fever in 13 patients (54.2%). Neurological deficits depend on abscess location. Brainstem abscesses tend to reveal cranial nerve palsy and disturbances in afferent and efferent long tracts, causing typically hemiparesis [36]. Cranial nerve palsy was diagnosed in 19 patients (79.2%) and hemiparesis in 16 patients (66.7%). The most commonly affected nerves were facial (VII) and abducens (VI). However, all cranial nerve palsies were identified in pediatric brainstem abscess patients, except for the hypoglossal nerve (XII). Altered level of consciousness was found out in 10 patients (41.7%). All these patients presented with somnolence; accordingly, there was no incidence of coma at all. Typically, 30–50% of pediatric patients diagnosed with brain abscess present with seizures [37]. Notwithstanding, considering brainstem abscesses, an analysis revealed only one patient with a medical history of abscess-related seizures, accounting for just 4.2% of the entire group (Table 2).

## Diagnosis

### Neuroimaging

Brain imaging techniques, including MRI or CT scans, are crucial in a diagnostic process. Therefore, they should always be performed when suspicion of brain abscess exists. Since the introduction of the CT scan in 1974, the overall brain abscess–related mortality decreased from 40 to 20% [31]. Computed tomography allows localizing and identifying a number of abscesses, assessing cerebritis area, and mass effect; however, we cannot discriminate between abscess and tumor. In contrast, MRI allows making a differential diagnosis with different lesions and is associated with less toxicity and better resolution [32, 38]. MRI supplied by diffusion-weighted imaging (DWI) allows differentiating abscess from cystic or necrotic lesions. It appears as a hyperintense mass in DWI and hypointense in apparent diffusion coefficient (ADC) as a result of restricted diffusion through the abscess capsule. Sensitivity and specificity for DWI in differentiating brain abscess from other cystic lesions are estimated at 96% [39]. Therefore, many authors state that in the case of brain abscess suspicion, the MRI scan should be the imaging of choice [32, 33, 38]. CT or MRI abscess features depend on the infection phase and etiology of infection [40]. Typically, it appears hypointense on CT and T1WI MRI and hyperintense on T2WI.

Both, contrast CT- and gadolinium-enhanced MRI, reveal characteristic, regular ring enhancement, which is present at the periphery of the lesion. Usually, significant cerebritis is visible in the surrounding of the abscess, on both CT and MRI scans. Magnetic resonance spectroscopy (MRS), which enables the discovery of lesion’s metabolic profile, seems to be a very promising sequence in the diagnostic process of brain abscess. A combination of DWI with MRS can improve the specificity and sensitivity of abscess diagnosis from 61% and 62%, respectively, to 100% for both parameters [41]. Diffusion tensor imaging (DTI) can provide essential measures, significantly differentiating abscesses from abscess-mimicking glioblastomas or metastases [42]. Those modalities can be particularly helpful in establishing a diagnosis of an abscess when a clinical presentation does not clearly indicate an abscess. However, the differential diagnosis should always include high-grade glioma, whose appearance can be misleading even in DWI [43]. To date, 11 brainstem abscesses were diagnosed based on CT-scan features and eight basing on MRI scan. The remaining five abscesses were visualized via pneumoencephalography just before the CT scan introduction in 1974.

### Laboratory findings and microbiology

Evaluation of blood CRP level, erythrocyte sedimentation rate (ESR), and leukocyte count could promptly indicate if pathologic mass in the brain has an infectious character. Nevertheless, inflammatory parameters can be within normal ranges in up to 30–40% of patients; therefore, blood tests cannot rule out abscess [44]. Although they do not provide any additional value in the diagnosis of brain abscess, they should routinely be included in the primal diagnostic process.

Lumbar puncture should be performed occasionally and with clear indications because of the potential risk of brain herniation. The elevated opening pressure is observed in 70% of patients [45]. Some authors state that lumbar puncture is contraindicated in suspicion of brain abscess [6]. Moreover, CSF cultures in children are usually negative unless abscess ruptured into the ventricular system, or meningitis coexists [29, 36]. It is worth mentioning that abscesses rupture promptly result in elevated intracranial pressure; therefore, lumbar puncture should be contraindicated in those cases, too [45]. Papilledema was observed on admission in two (8.3%) pediatric patients with brainstem abscess, indicating intracranial hypertension.

Furthermore, three additional patients had hydrocephalus (12.5%), which was present generally in four children (16.7%). Depicted analysis indicates that in 20.8% of children with brainstem abscess diagnosis, a lumbar puncture was initially contraindicated. CSF obtained via lumbar puncture may show mononuclear pleocytosis, protein elevation, and normal glucose levels. However, these parameters can be within normal limits in up to 16% of patients [31].

Identification of causative pathogens is crucial for the implementation of targeted antimicrobial treatment. Pathogen type depends on infection source, patient age, and underlying comorbidities [37]. They are isolated in about two-thirds of patients who did not undergo antimicrobial therapy before material collection. In contrast, patients who underwent antibiotic treatment preoperatively showed positive pus cultures in merely 35% [46]. According to previous studies, CSF cultures or blood cultures are rarely positive comparing with pus microbiology. Blood cultures are positive in 2.8–28.6%; however, studies regarding this issue are inconsistent. CSF utility is also very limited, ranging from 2.8 to 44% of positive cultures [32].

Nevertheless, meta-analysis provided by Brouwer et al. indicated that 28% of CSF cultures and 24% of blood cultures are positive [31]. In 24 described brainstem abscesses, neither CSF nor blood was helpful in the identification of microorganisms in any patient, comparing to 61.1% of positive pus cultures. Those discrepancies could be caused by a considerably lower number of performed CSF and blood microbiological tests comparing to the number of pus tests. Laboratory findings of CSF, blood, and pus are described in Table 3.

**Table 3:** Laboratory findings and microbiology of pediatric patients with brainstem abscess [5–26]

Features	Number (%)
Microbiological tests	22/24 (91.7)
Positive pus cultures	11/18 (61.1)
Positive CSF cultures	0/4
Positive blood cultures	0/4
Monomicrobial abscess	10/11 (90.9)
Polymicrobial abscess	1/11 (9.1)
Pus microorganisms	12
*Streptococcus* spp.	4/12 (33.3)
*Staphylococcus* spp.	3/12 (25.0)
*Peptostreptococcus* spp.	3/12 (25.0)
*Fusobacterium* spp.	1/12 (8.3)
*Haemophilus* spp.	1/12 (8.3)
CSF tests	14/24 (58.3)
Normal	4/14 (28.6)
Leukocytosis	7/14 (50.0)
Elevated protein	5/14 (35.7)
Normal glucose level	13/14 (92.9)
Blood tests	12/24 (50.0)
Normal	3/12 (25.0)
Leukocytosis	8/12 (66.7)
Elevated CRP level	1/12 (8.3)
High ESR	2/12 (16.7)

Most abscesses are monomicrobial, although about 25% can be polymicrobial, which is said to be associated mainly with otogenic infection source [29, 31]. Our analysis revealed only one positive polymicrobial abscess, however unrelated to otitis. The distribution of microorganisms is generally equal in adults and children [31]. The majority of authors state that Gram-positive cocci (Streptococci and Staphylococci) and anaerobes are the most frequently isolated pus microorganisms [27, 47]. Brouwer et al. indicated a higher incidence of Gram-negative enteric bacteria than anaerobes.

Nevertheless, these differences were not considerable; therefore, both groups of microorganisms should be considered to have comparable incidence [31]. In brainstem abscesses, Gram-positive cocci (Streptococci and Staphylococci) dominated, encompassing 58.3% of identified pathogens, followed by 33.3% of anaerobes and 8.3% of Gram-negative non-enteric bacteria (Table 3).

The identification of infection source seems to be crucial in the implementation of efficient therapy. In that way, we can presume which group of bacteria is likely to be present in the brain abscess.

That knowledge allows applying the most accurate empiric antibiotic therapy, therefore increasing the probability of successful treatment without the necessity of neurosurgical abscess drainage. Streptococci being the most frequently appearing bacteria spread through hematogenous route from distant infection sources such as endocarditis or other cardiac-related diseases [37]. Moreover, Streptococci, with a particular impact on *Streptococcus intermedius*, form the physiological flora of the oral cavity and upper respiratory tract. Thus, cerebral abscesses, including Streptococci, are likely to originate from continuous infection from otitis, sinusitis, or dental source [48]. It is said that cyanotic congenital heart disease is a significant risk factor for *S. intermedius* abscess development due to the formation of microinfarcts and the increase of blood-brain barrier permeability. Cardiogenic etiology accounts for about 25–46% of *S. intermedius*–related brain abscesses [30].

Moreover, according to our analysis, cardiac diseases were the most common etiologic factors for brainstem abscess in the pediatric population, forming 25% (Table 2). Staphylococci, the second most common pathogens, typically occur in abscesses resulting from trauma, neurosurgical procedure, or also in association with endocarditis [33]. Anaerobes can be found when pyogenic pulmonary disease, dental infections, or sinusitis are identified. Analysis of brainstem abscesses shows that hematogenous spread from cardiogenic infection source was positive for Peptostreptococci twice and Streptococci once. Otitis resulted in *Fusobacterium* isolation, meningitis was associated with *Staphylococcus*, and finally, dental source resulted in *Haemophilus paraprophilus*–positive cultures. The available literature, including our patient, suggests that 38.9% of brainstem lesions are sterile. The majority of studies suggest the overall 30% incidence of sterile pediatric brain abscesses [27, 29].

### Management

A limited number of pediatric patients diagnosed with brainstem abscess preclude the establishment of treatment guidelines. Successful management should include the identification of the infection source and its intensive therapy. Theoretically, neurosurgical aspiration is superior to antibiotics only. It allows assessing microbiology of the lesion, additionally reduces the number of pathogens and disrupts abscess capsule. Better penetration of antibiotics through an impaired capsule leads to an increase in their concentration, improving the efficacy and length of antimicrobial treatment. Furthermore, neurosurgical intervention enables immediate relief of mass effect by reducing abscess volume [49]. Due to the high incidence of a focal neurologic deficit on admission (87.5%), surgical aspiration aiming to reduce mass effect exerted on brainstem structures should always be considered to gain an immediate improvement in the patient’s neurologic condition. Moreover, the location close to the ventricular system poses a threat of intraventricular rupture with subsequent fatal complications [2]. According to recommendations proposed by Arlotti et al., patients who present with GCS > 12, small, at least 2.5-cm abscess diameter, multiple abscesses, or with culture obtained from other sources (CSF/blood) should be considered for antibiotic therapy only [50]. Jamjoom suggested that children in stable clinical condition and with the availability of neuroimaging control should be treated conservatively [16]. Furthermore, the majority of authors state that surgical intervention should be performed as soon as clinical deterioration occurs or features of abscess enlargement manifest on CT scan [5, 16, 51]. Most children with brainstem abscess were treated with a standard craniotomy (14 patients, 58.3%), six (25%) were given antibiotics only, and the remaining four patients (16.7%) underwent stereotactic abscess aspiration (Table 4). No surgery-related complications were observed in both standard craniotomy and stereotactic aspiration. Due to satisfactory outcomes, safety, and immediate improvement of the patient’s clinical state, neurosurgical intervention should always be considered.

**Table 4:** Treatment options and outcomes in pediatric patients with brainstem abscess [5–26]

Feature	Number (%)
Treatment	
Conservative only	6/24 (25.0)s
Aspiration via craniotomy	14/24 (58.3)
Burr-hole stereotactic aspiration	4/24 (16.7)
Outcomes	
Complete neurological recovery	8/24 (33.3)
Improved with minor deficits	11/24 (45.8)
Unchanged	2/24 (8.3)
Worsened	0
Died	3/24 (12.5)

There is no evidence supporting the dominance of one surgical technique in the treatment of brainstem abscess in children. On the other hand, stereotactic or neuronavigation-assisted surgery is said to be safer and more beneficial than the open craniotomy, even when the diagnosis of brainstem abscess is not entirely established preoperatively [22]. These minimally invasive techniques allow both to assess the character of the lesion via biopsy and to perform aspiration of the pus when an abscess is confirmed. Still, these are no risk-free methods because bleeding from the abscess capsule can always occur. Further studies need to be conducted to assess the most efficient surgical technique in these patients. Empirical antibiotics should be implemented as soon as a diagnosis of a cerebral abscess is established and should include broad-spectrum intravenously administered antibiotics with good penetration through the blood-brain barrier [47]. Antibiotics are advised for 6–8 weeks of treatment time; however, theoretically, they should be administered until the abscess cavity disappears on brain MRI. Empirical therapy should cover Gram-positive, Gram-negative bacteria, and anaerobes; therefore, it is indicated to use broad-spectrum cephalosporins with metronidazole [44]. Therapy should be modified after obtaining a positive culture, according to causative microorganisms and antibiograms.

### Prognosis and outcomes

Despite advances in diagnosis and therapy of cerebral abscesses, they remain a serious disease associated with high morbidity [36]. Currently, the mortality rate for brain abscesses is estimated at 5–15% values and has been decreasing to that level since the CT scan was introduced in 1974 [35]. In a recent analysis of brainstem abscesses, mortality was estimated at 12.5%, based on data concerning three pediatric patients diagnosed in 1974, 1975, and 1976. Improvement was noted in 79.1% of patients, with complete resolution of symptoms in 33.3% of patients. Children who did not make a complete neurological recovery comprised 45.8% of the overall group (Table 4). Literature suggests that no neurologic deficits or just minor sequelae are described in 70% of patients with cerebral abscesses [44]. No patient with significant disabilities on follow-up was described. To sum up, the prognosis for brainstem abscess is good, being similar to the entire group of cerebral abscesses.

### Exemplary case description

**Fig. 1:**
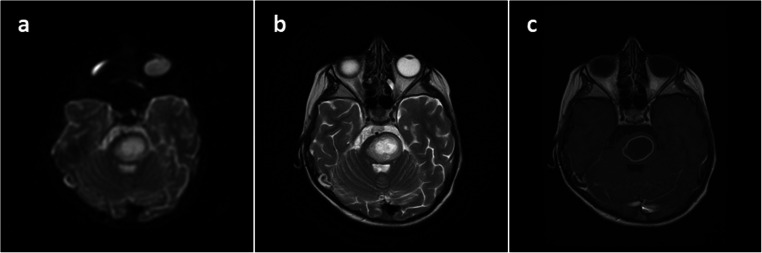
Axial cranial MRI **a** DWI, **b** T2-weighted, and **c** T1-weighted without gadolinium showing pontine abscess

**Fig. 2:**
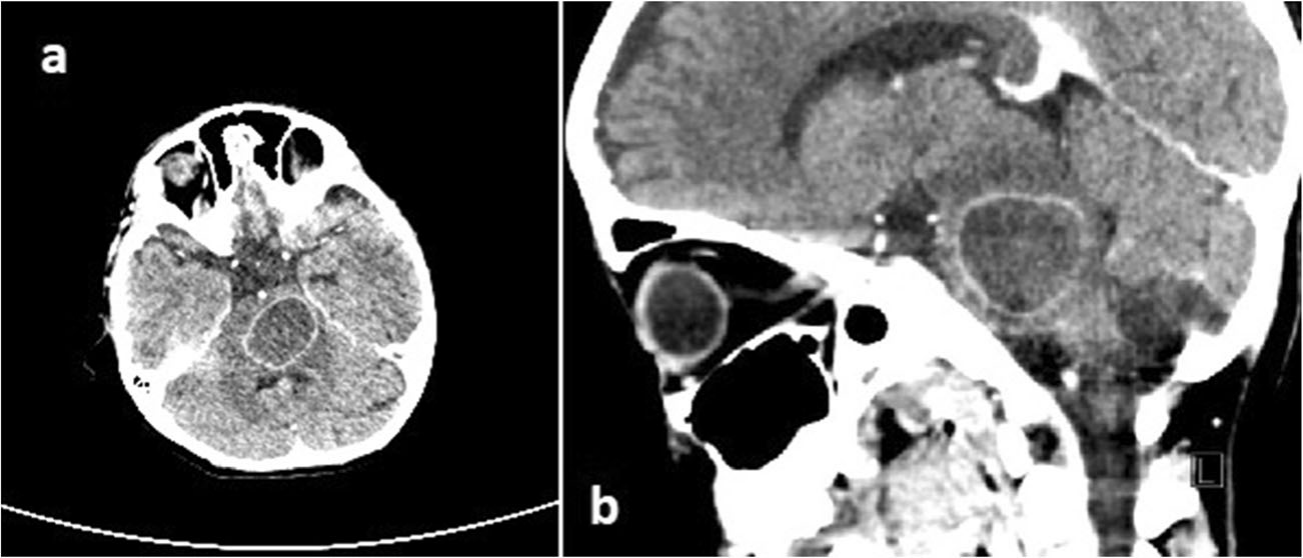
CT with contrast **a** axial and **b** sagittal reconstruction showing enlargement of the abscess (performed before biopsy)

A 5-year-old girl was admitted to a local pediatric department with fever and gait disturbances. The patient was somnolent with negative meningeal signs, nasopharyngeal cavity infection, and labial herpes noticed on examination. Inflammation markers were not elevated. Acyclovir was administered due to suspected herpes encephalitis. On the admission day, significant deterioration occurred with positive meningeal signs, tetraparesis, and consciousness disturbances. Computed tomography (CT) and subsequent MRI revealed hypodense, rim-enhanced mass located in the pons with restriction of diffusion on DWI-weighted imaging (Fig. 1). Inflammation markers were still within the normal range; elevated cytosis was noticed in the CSF test. The patient was given ceftriaxone, vancomycin, and steroids; acyclovir was left off. After that, she improved clinically; however, right-sided paresis was still severe. A slight improvement was observed within a few days of intensive therapy, although the patient remained without verbal contact. No changes in abscess measurements were noticed on the control MRI scan. A few days later, significant deterioration occurred with high fever, tachypnea, anisocoria, saturation < 90%, and pulse variations 50–180/min. Urgent CT scan revealed enlargement of pathologic mass with surrounding edema (Fig. 2). On the next day, the patient was taken to the operating room for navigated abscess puncture. Posterior fossa entry-point through the middle cerebellar peduncle was chosen, and then 10 cm^3^ of pus was evacuated from the abscess. Postsurgical CT showed a decrease in abscess volume. The patient was still without contact, with severe right-sided paresis and anisocoria.

**Fig. 3:**
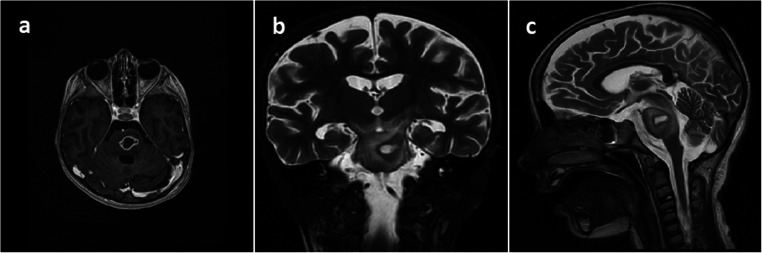
MRI performed 3 days after biopsy **a** horizontal with gadolinium, **b** frontal T2-weighted, and **c** sagittal T2-weighted showing reduction of abscess volume, but the enlargement of surrounding encephalitis area

Antibiotic therapy was continued. Another deterioration with the presence of severe tetraparesis was observed 3 days later. MRI scan revealed a decrease of abscess volume, but the surrounding encephalitis area enlarged (Fig. 3). Microbiological examination showed *Streptococcus intermedius* cultured from the pus. Meropenem was administered as a targeted antibiotic. Subsequently, a mild improvement was being observed over a few next days. The patient became conscious with still severe right-sided paresis, mild on the left side.

**Fig. 4:**
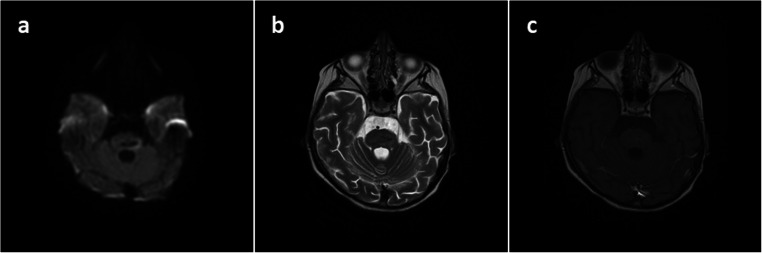
Axial cranial MRI **a** DWI, **b** T2-weighted, and **c** T1-weighted with gadolinium performed 1 month after admission showing nearly complete disappearance of the abscess

MRI scan performed 1 month after admission showed nearly complete disappearance of the abscess (Fig. 4). Subsequently, she was discharged with the recommendation of intensive rehabilitation. During the next weeks, further clinical improvement was observed. Now, the patient is fully conscious, talking, and walking with only mild right-sided hemiparesis.

## Conclusions

Brainstem abscesses are rare findings, with merely 24 pediatric patients being described in the literature so far. Neurologic appearance is typical for pathologic mass occupying the brainstem, presenting with cranial nerve palsy and long tracts impairment, typically in the form of hemiparesis. The combination of MRI spectroscopy with DWI findings would increase the specificity and sensitivity for brain abscess up to 100%. Neurosurgical aspiration is safe and beneficial and should always be considered and performed immediately when conservative therapy is not successful, and clinical deterioration occurs. It is worth mentioning that there is no evidence supporting the dominance of one surgical technique in the treatment of pediatric brainstem abscesses. Prognosis in pediatric brainstem abscess is generally favorable. Most patients recover with minor neurologic deficits or improve completely.
